# Comparable Ascertainment of Newly-Diagnosed Atrial Fibrillation Using Active Cohort Follow-Up versus Surveillance of Centers for Medicare and Medicaid Services in the Atherosclerosis Risk in Communities Study

**DOI:** 10.1371/journal.pone.0094321

**Published:** 2014-04-11

**Authors:** Lindsay G. S. Bengtson, Anna Kucharska-Newton, Lisa M. Wruck, Laura R. Loehr, Aaron R. Folsom, Lin Y. Chen, Wayne D. Rosamond, Sue Duval, Pamela L. Lutsey, Sally C. Stearns, Carla Sueta, Hsin-Chieh Yeh, Ervin Fox, Alvaro Alonso

**Affiliations:** 1 Division of Epidemiology and Community Health, University of Minnesota, Minneapolis, Minnesota, United States of America; 2 Department of Epidemiology, University of North Carolina Chapel Hill, Chapel Hill, North Carolina, United States of America; 3 Department of Biostatistics, University of North Carolina Chapel Hill, Chapel Hill, North Carolina, United States of America; 4 Division of Cardiology, University of Minnesota, Minneapolis, Minnesota, United States of America; 5 Department of Health Policy and Management, University of North Carolina Chapel Hill, Chapel Hill, North Carolina, United States of America; 6 Division of Cardiology, University of North Carolina Chapel Hill, Chapel Hill, North Carolina, United States of America; 7 Division of General Internal Medicine and Division of Epidemiology, Johns Hopkins University, Baltimore, Maryland, United States of America; 8 Department of Cardiology, University of Mississippi, Jackson, Mississippi, United States of America; University of Louisville, United States of America

## Abstract

**Objective:**

Increasingly, epidemiologic studies use administrative data to identify atrial fibrillation (AF). Capture of *incident* AF is not well documented. We examined incidence rates and concordance of AF diagnosis based on active cohort follow-up versus surveillance of Centers for Medicare and Medicaid Services data in the Atherosclerosis Risk in Communities study.

**Methods:**

Atherosclerosis Risk in Communities cohort participants without prevalent AF enrolled in fee-for-service Medicare, with inpatient and outpatient coverage, for at least 12 continuous months between 1991 and 2009 were included. In active Atherosclerosis Risk in Communities study follow-up, annual telephone calls captured hospitalizations and deaths with incident AF diagnosis codes. For Centers for Medicare and Medicaid Services data, incident AF was defined by billed inpatient and outpatient diagnoses.

**Results:**

Of 10,134 eligible cohort participants, 738 developed AF according to both Atherosclerosis Risk in Communities and Centers for Medicare and Medicaid Services data; an additional 93 and 288 incident cases were identified using only Atherosclerosis Risk in Communities and Centers for Medicare and Medicaid Services data, respectively. Incidence rates per 1,000 person-years were 10.8 (95% confidence interval: 10.1–11.6) and 13.6 (95% confidence interval: 12.8–14.4) in Atherosclerosis Risk in Communities and Centers for Medicare and Medicaid Services, respectively; agreement was 96%; kappa was 0.77 (95% confidence interval: 0.75–0.80). Earlier AF ascertainment by one system versus the other was not associated with any cardiovascular disease risk factors, after accounting for sociodemographic factors. Additional Centers for Medicare and Medicaid Services events did not alter observed associations between risk factors and AF.

**Conclusion:**

Among fee-for-service enrollees, AF incidence rates were slightly lower for active cohort follow-up than for Centers for Medicare and Medicaid Services surveillance, because the latter included outpatient atrial fibrillation. Concordance was high and combining the two approaches could provide a more complete picture of newly-diagnosed AF.

## Introduction

Increasingly, administrative data are used for research purposes, including epidemiologic studies to identify patients with cardiovascular diseases (CVDs) [1]–[5] such as atrial fibrillation (AF) [6]. The ability to efficiently and inexpensively access information on a large number of people makes administrative claims an appealing source of outcomes for epidemiologic research. However, the usefulness of this approach varies by numerous factors, including the disease algorithm chosen and the population studied. Medicare data are often used but are limited to those ≥65 and not having supplemental health maintenance organization (HMO) coverage. High-performing algorithms have been developed to identify major CVDs [1]–[5]. A recent systematic review of algorithms used to identify AF in administrative data reported a median positive predictive value (PPV) of 89% (range: 70%–96%) and a median sensitivity of 79% (range: 57%–95%) [7].

Despite performance measures indicating that administrative data could be a promising source for identifying AF patients, gaps exist concerning the appropriateness of this approach. A systematic review of 16 unique studies found that only one examined the ability of administrative data to identify *incident* AF. In this single study, a physician reviewed a sample of 125 hospital discharge summaries with a first ICD-9 code for AF and ECGs performed during that hospitalization to determine the validity of using hospital discharge codes; the PPV for any AF was 89% and for incident AF was 62% [8]. No study has compared AF ascertainment using only inpatient or only outpatient claims compared to using both types of claims. An important limitation of some cohort studies, including the Atherosclerosis Risk in Communities (ARIC) and Cardiovascular Health Study (CHS) cohorts, is reliance exclusively on inpatient claims to identify AF [8], [9], which could result in under-ascertainment. Furthermore, the majority of studies were performed in predominately white populations. The validity of utilizing administrative data may vary by race/ethnicity, as one study performed a subgroup analysis among stroke patients and reported a lower sensitivity of AF ascertainment for blacks compared to whites [8].

We sought to address the problem of limited knowledge regarding the usefulness of administrative data to identify incident AF, the lack of inpatient and outpatient claims comparison, and the paucity of data in nonwhite populations. We compared overall and race-specific incidence rates of AF using the active ARIC follow-up method with surveillance of Centers for Medicare and Medicaid Services (CMS) administrative Medicare claims data (inpatient only, outpatient only and both inpatient and outpatient claims). We assessed concordance of AF diagnosis between the data sources and performed a descriptive analysis to identify factors associated with earlier diagnosis as well as concordance.

## Methods

### Ethics statement

The ARIC study has been approved by the Institutional Review Boards (IRB) of all participating institutions, including the IRBs of the University of Minnesota, Johns Hopkins University, University of North Carolina, University of Mississippi Medical Center, and Wake Forest University. Participants provided written informed consent during each of the ARIC study visits, including permission to utilize information derived from their healthcare utilization.

### Data sources

The ARIC study is a population-based biracial cohort of 15,792 participants aged 45–64 years at enrollment (1987–1989), from four communities in North Carolina, Mississippi, Minnesota, and Maryland [10]. Additional study exams occurred during three follow-up visits as well as annual telephone contact to obtain information about all hospitalizations and vital status, details of which have been reported previously [8].

The ARIC study has an Interagency Agreement with CMS to obtain Medicare data for ARIC cohort participants. Participants are matched on social security number, sex, and date of birth. Of the 15,738 ARIC participants alive on January 1, 1991, 14,530 (92.3%) were matched successfully and linked to CMS claims. Matched participants are linked to inpatient, outpatient, and carrier files. The Medicare Provider Analysis and Review (MedPAR) file contains claims for inpatient services covered under Medicare Part A. The outpatient files contain claims for services covered under Medicare Part B, including institutional claims (Outpatient file) for outpatient services and noninstitutional physician claims (Carrier file). Inpatient and outpatient CMS claims have been available for research since 1991.

### Study sample

**Figure 1:**
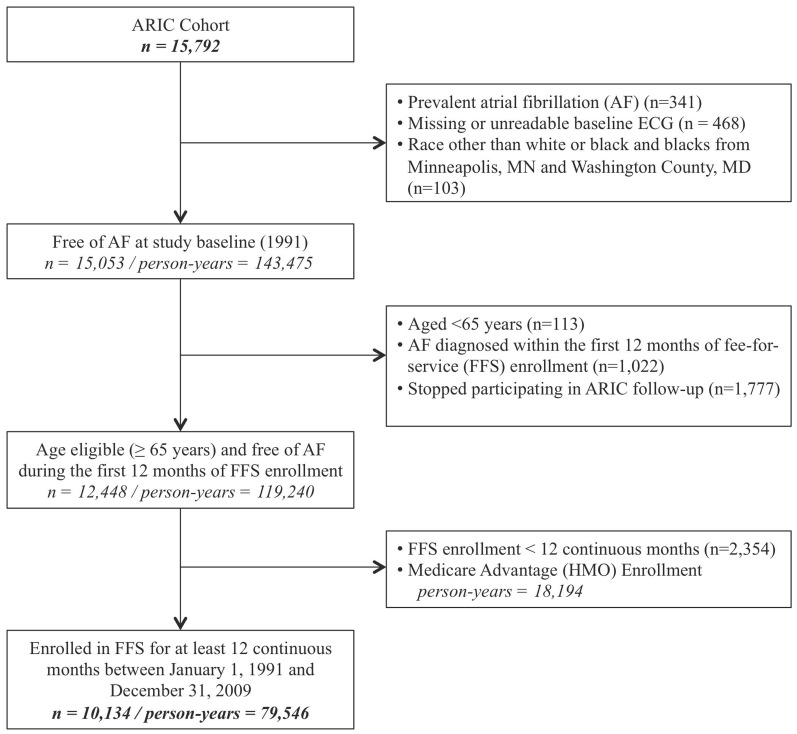
Flowchart of selection of study sample from the Atherosclerosis Risk in Communities Study. ARIC =  Atherosclerosis Risk in Communities. ECG =  Electrocardiogram. Participants were excluded if they met one or more of the exclusion criterion.

ARIC cohort participants enrolled in fee-for-service (FFS) Medicare, both Parts A and B, for at least 12 continuous months between January 1, 1991, and December 31, 2009, were eligible for inclusion (Figure 1). Fee-for-service enrollment was necessary because Medicare Advantage insurance plans are not required to submit claims for beneficiaries and those enrolled in only Part A have incomplete claims data. Participants whose race was not white or black and nonwhites from the Minneapolis and Washington County field centers were excluded due to small numbers. Those with missing or unreadable electrocardiograms (ECG) or prevalent AF at the initial ARIC study exam were excluded. In order to ascertain incident diagnoses, and remove prevalent cases of AF, participants with AF diagnosed during the first year of FFS enrollment, from either ARIC or CMS, were excluded, as were those with AF diagnosed based on ARIC data before January 1, 1992 because CMS data are available for research beginning January 1, 1991. Participants enrolled in Medicare due to disability or certain covered medical conditions were not included in the study unless they met eligibility criteria after becoming age eligible (aged ≥65 years) for Medicare enrollment.

### Definition of atrial fibrillation

Active ARIC follow-up identifies AF cases through three sources: study visit ECGs, hospital discharge codes, and death certificates [8]. AF cases obtained exclusively from study ECGs (n = 4) were not included as AF events due to their subclinical nature and to ensure consistent methods of ascertainment between data sources. For this analysis, incident cases of AF were ascertained from January 1, 1991, through December 31, 2009, through two sources: hospital discharge codes, *International Classification of Diseases, 9^th^ revision*, (ICD-9) codes 427.3, 427.31 or 427.32, in any position, and death certificates with *International Classification of Diseases, 10^th^ revision* (ICD-10) code I48 or ICD-9 code 427.3x as the underlying cause of death. The AF incidence date was defined as the date of first hospital discharge with an AF diagnosis, or death by AF, whichever occurred earlier.

For MedPAR and outpatient CMS claims, incident AF was defined as an AF discharge diagnosis (ICD-9 code 427.3, 427.31 or 427.32), in any position, on a single inpatient claim or as a diagnostic code on two outpatient claims within 7–365 days. A minimum of two outpatient claims at least 7 days apart were required to reduce the likelihood of including “rule out” diagnoses and to improve algorithm specificity [6], [11]. The AF incidence date was defined as the discharge date for a MedPAR short-stay claim or the date of the second qualifying outpatient claim, whichever occurred earlier. Secondary CMS AF definitions were restricted to only MedPAR claims criteria and only outpatient claims criteria.

Atrial fibrillation following cardiac operative procedures is fairly common [12]; in both active ARIC follow-up and CMS surveillance, an AF diagnosis occurring simultaneously with cardiac revascularization (ICD-9 code 36.X) or other cardiac surgery involving heart valves or septa (ICD-9 code 35.X) during the index hospitalization, without a subsequent diagnosis of AF, was not considered an AF diagnosis.

### Assessment of covariates

During the baseline ARIC study exam (1987–1989), standardized methods were used to collect data on age, race, sex, educational achievement, cigarette smoking, ethanol consumption, height, weight, blood pressure, antihypertensive medication use, diabetes mellitus, total cholesterol, low-density lipoprotein cholesterol, high-density lipoprotein cholesterol, triglycerides, and previous myocardial infarction, heart failure or coronary heart disease [10]. An ECG Cornell voltage score >28 mm in men or >22 mm in women was considered evidence of left ventricular hypertrophy [13]. Baseline age is age upon meeting enrollment criteria for the present analysis. All other characteristics are from the baseline ARIC study exam.

### Statistical analysis

Person-years of follow-up were calculated as the date of study eligibility, following 12 months of continuous enrollment in FFS without an AF diagnosis, to the date of AF diagnosis, death, loss to follow-up, cessation of FFS enrollment, or December 31, 2009, whichever occurred earliest. Person-years of follow-up were attributed to age- (5 year age groups), sex- and race- (whites and blacks) specific groups. Age-, sex- and race-specific rates were calculated dividing the number of incident AF cases by the corresponding person-years of follow-up. Age- and sex-standardized rates of incident AF for whites and blacks separately were calculated using the sex and age (65–69 years, 70–74 years, 75–79 years and 80 years and older) person-time distribution of the eligible cohort.

Concordance of incident AF events between data sources was assessed with Cohen's Kappa (Κ) statistic, a chance-adjusted measure of agreement [14]. Percent agreement, overall, as well as positive and negative agreement, were calculated [15]–[17].

A descriptive analysis, restricted to participants with incident AF ascertained in both ARIC and CMS, and with complete covariate data, was performed to determine the mean difference in incident date. Linear regression, with time between diagnosis dates in ARIC and CMS, was used to determine predictors of earlier diagnosis. Log-binomial regression, restricted to participants with AF ascertained from at least one data source and with complete covariate data, was used to identify demographic and clinical factors associated with concordance. Age, sex and a composite race and center variable were retained in the linear and log-binomial regression models regardless of statistical significance. Cox proportional hazards regression was used to determine the association between established risk factors and incident AF based exclusively on active ARIC follow-up; subsequently, cases of incident AF ascertained only from surveillance of CMS data were included to determine the impact of these additional events on the associations. All statistical analyses were performed with SAS, version 9.2 (SAS Institute, Cary, NC).

## Results

Of the original 15,792 ARIC participants, our final sample included 10,134 participants who were initially free of AF and enrolled in FFS for at least 12 continuous months between January 1, 1991 and December 31, 2009. Notably, 18,194 person-years available to ARIC had to be omitted because those participants were in Medicare Advantage and therefore had incomplete CMS claims (Figure 1). Active ARIC follow-up ascertained 831 incident AF diagnoses during 76,754 person-years of follow-up. The corresponding figures from combined inpatient and outpatient CMS surveillance were 1,026 (considering inpatient and outpatient ascertainment separately: 736 unique inpatient and 827 unique outpatient) AF diagnoses during a total of 75,596 (considering inpatient and outpatient follow-up separately: 76,887 inpatient and 76,293 outpatient) person-years of follow-up. Baseline characteristics of the study sample stratified by source of AF diagnosis are shown in Table 1. Overall, the mean age at baseline, date of study eligibility, was 66.4 years (standard deviation 1.5 years) and women accounted for slightly over half and blacks for a quarter of the study sample. Among AF diagnoses ascertained exclusively by active ARIC follow-up, 32% were among blacks, while among those ascertained only in CMS surveillance, 13% were among blacks. Participants with AF diagnosed from both data sources had a higher prevalence of prior myocardial infarction and heart failure compared to those with AF ascertained from only one source.

**Table 1:** Baseline* (1987–89) characteristics of Atherosclerosis Risk in Communities participants enrolled in Medicare fee-for-service, overall and by source of incident atrial fibrillation diagnosis.

	Total	No Incident AF Diagnosis	ARIC Only AF Diagnosis	CMS Only AF Diagnosis	ARIC and CMS AF Diagnosis	
	(n = 10,134)	(n = 9015)	(n = 93)	(n = 288)	(n = 738)	p-value
Age, years	66.4±1.5	66.4±1.5	66.7±1.9	66.5±1.6	66.6±1.6	0.01
Women, %	57.0	58.5	46.2	42.4	45.4	<0.0001
Black, %	26.0	27.2	32.3	13.2	15.3	<0.0001
High school graduate, %	76.8	77.4	62.4	76.4	71.5	<0.0001
Current smoker, %	23.2	22.6	30.1	22.2	29.3	0.0002
Current drinker, %	55.3	55.3	46.7	57.6	55.0	0.34
BMI (kg/m^2^)	27.7±5.2	27.6±5.2	28.8±5.6	28.1±5.1	28.6±5.4	<0.0001
Hypertension, %	34.2	32.8	39.8	40.0	49.0	<0.0001
Antihypertensive medication, %	25.0	23.8	25.0	31.9	37.1	<0.0001
Diabetes mellitus, %	10.6	10.0	18.3	12.9	16.4	<0.0001
Total cholesterol, mg/dL	216.6±41.7	216.3±41.6	220.8±49.6	217.2±45.4	219.3±39.5	0.20
LDL-c, mg/dL	139.0±39.2	138.5±39.2	143.6±47.4	141.8±44.0	142.8±35.6	0.01
HDL-c, mg/dL	52.1±17.0	52.5±17.1	49.1±14.5	49.4±16.4	47.7±15.6	<0.0001
Triglycerides, mg/dL	130.7±85.3	129.0±84.1	140.5±71.5	136.2±100.9	147.8±92.0	<0.0001
Left ventricular hypertrophy, %	1.8	1.6	2.2	2.9	3.7	0.0002
Previous myocardial infarction, %	3.4	2.8	3.2	6.6	9.5	<0.0001
Heart failure, %	4.1	3.7	4.4	4.3	8.5	<0.0001
Coronary heart disease, %	4.1	3.4	3.3	9.1	11.4	<0.0001

*Baseline age is age upon meeting enrollment criteria for the present analysis. All other characteristics are from the initial ARIC study exam (1987–1989).

ARIC =  Atherosclerosis Risk in Communities.

CMS =  Centers for Medicare and Medicaid Services.

Continuous variables presented as mean ± standard deviation (SD).

P-values from testing the null hypothesis of independence from chi-square (categorical) and F-test (continuous).

**Figure 2:**
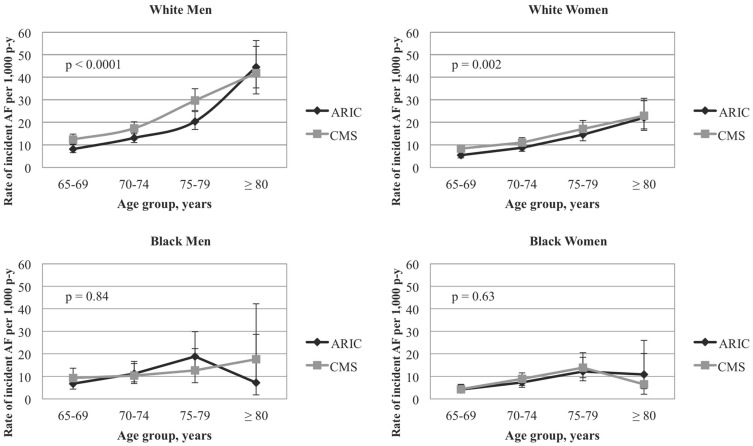
Age-, sex- and race-specific incidence rates of atrial fibrillation by source of diagnosis. CMS includes inpatient (MedPAR) or outpatient diagnosis of atrial fibrillation. 1,000 p-y = 1,000 person-years. Vertical bars represent 95% confidence intervals. P-values from testing the null hypothesis that the incidence rate ratio for each sex and race group (ARIC compared to CMS) equals one.

The age-, sex- and race-specific incidence rates were slightly higher based on CMS ascertainment of AF but followed a pattern similar to the rates based on active ARIC follow-up (Figure 2). Among participants with AF diagnosed in both data sources, 63% had identical dates of AF diagnosis from ARIC and CMS and nearly 75% had diagnoses within ±30 days. Earlier ascertainment of AF by one system versus the other was not associated with any cardiovascular disease risk factors, after accounting for sociodemographic factors. After accounting for differences in the age and sex distribution of whites and blacks by standardizing the rates to the study sample (Table 2), the AF incidence rate based on ARIC ascertainment, per 1,000 person- years, was 11.4 (95% confidence interval (CI): 10.5–12.2) and 8.6 (95% CI: 7.1–10.0) among whites and blacks, respectively. The comparable rates from CMS surveillance of AF were 14.8 (95% CI: 13.8–15.8) and 8.9 (95% CI: 7.5–10.4) for whites and blacks, respectively. Using secondary CMS AF definitions, restricted to inpatient claims criteria, the corresponding rates were 10.3 (95% CI: 9.5–11.1) and 6.6 (95% CI: 5.3–7.8), among whites and blacks, respectively; restricted to outpatient claims criteria, the rates were 12.1 (95% CI: 11.2–13.0) for whites and 6.4 (95% CI: 5.1–7.6) for blacks. Utilizing the secondary CMS definition of AF, restricted to inpatient claims criteria, among participants with AF diagnosed in both sources, 90% of participants had AF diagnosed on the same day and 93% were within ±30 days. When considering only outpatient claims criteria for CMS surveillance of AF compared to active ARIC follow-up, among participants with AF diagnosed in both sources, 61% of AF diagnoses occurred earlier in ARIC (hospital discharge date) compared to outpatient CMS surveillance.

**Table 2:** Race-specific incidence rates of atrial fibrillation among Atherosclerosis Risk in Communities participants enrolled in Medicare fee-for-service by source of diagnosis.

Source of Diagnosis	Whites (n = 7504)	Blacks (n = 2630)	p-value
**ARIC Data**			
Incident AF (n/person**-**years)	688/58606.8	143/18146.7	
Unadjusted incidence rate*	11.7 (10.9–12.7)	7.9 (6.7–9.3)	<0.0001
Age- and sex-standardized incidence rate*	11.4 (10.5–12.2)	8.6 (7.1–10.0)	0.003
**All CMS Data** †			
Incident AF (n/person**-**years)	875/57528.8	151/18067.6	
Unadjusted incidence rate*	15.2 (14.2–16.3)	8.4 (7.1–9.8)	<0.0001
Age- and sex-standardized incidence rate*	14.8 (13.8–15.8)	8.9 (7.5–10.4)	<0.0001
**Inpatient (MedPAR) Data**			
Incident AF (n/person**-**years)	623/58677.7	113/18209.6	
Unadjusted incidence rate*	10.6 (9.8–11.5)	6.2 (5.2–7.5)	<0.0001
Age- and sex-standardized incidence rate*	10.3 (9.5–11.1)	6.6 (5.3–7.8)	<0.0001
**Outpatient CMS Data**			
Incident AF (n/person**-**years)	721/58094.2	106/18198.5	
Unadjusted incidence rate*	12.4 (11.5–13.4)	5.8 (4.8–7.0)	<0.0001
Age- and sex-standardized incidence rate*	12.1 (11.2–13.0)	6.4 (5.1–7.6)	<0.0001

ARIC =  Atherosclerosis Risk in Communities.

CMS =  Centers for Medicare and Medicaid Services.

*Rates per 1,000 person-years (95% confidence intervals).

† Includes inpatient (MedPAR) and outpatient diagnosis of atrial fibrillation.

P-values from testing the null hypothesis that the incidence rate ratio (whites compared to blacks) equals one.

Cohen's Κ for concordance of incident AF diagnosis between ARIC cohort follow-up and CMS data was 0.77 (95% CI: 0.75–0.80) (Table 3). Comparing inpatient ascertainment of AF, the primary method of AF detection in ARIC, the Κ statistic improved to 0.85 (95% CI: 0.83–0.87). Race-specific Κ statistics were similar to the overall sample estimates (not shown) except for active ARIC follow-up versus CMS outpatient surveillance; concordance was lower among blacks, 0.56 (95% CI: 0.48–0.63). After accounting for age, sex and race/center, a descriptive analysis did not identify any factors associated with concordance between data sources.

**Table 3:** Overall concordance of incident atrial fibrillation diagnosis based on Atherosclerosis Risk in Communities data and Centers for Medicare and Medicaid Services data.

		All CMS*			Inpatient (MedPAR) CMS			Outpatient CMS		
		AF	No AF	Total	AF	No AF	Total	AF	No AF	Total
ARIC Cohort Follow-up	AF	738	93	831	673	158	831	563	268	831
	No AF	288	9015	9303	63	9240	9303	264	9039	9303
	Total	1026	9108	10134	736	9398	10134	827	9307	10134
Kappa 95% confidence interval		0.77 (0.75–0.80)			0.85 (0.83–0.87)			0.65 (0.62–0.68)		
% agreement		96			98			95		
% positive agreement		66			75			51		
% negative agreement		96			98			94		

ARIC =  Atherosclerosis Risk in Communities.

CMS =  Centers for Medicare and Medicaid Services.

*All CMS includes MedPAR and outpatient claims.

Inpatient CMS includes MedPAR claims.

Outpatient CMS includes outpatient and carrier claims.

% agreement calculated as the number of participants with consistent classification of diagnosed AF from active ARIC cohort follow-up and surveillance of CMS divided by the total number of observations and converted to a percent.

% positive agreement calculated as the number of participants classified as having AF based on both active ARIC cohort follow-up and surveillance of CMS, conditional on being classified as having AF from at least one source, and converted to a percent.

% negative agreement calculated as the number of participants classified as not having AF based on both active ARIC cohort follow-up and surveillance of CMS, conditional on being classified as not having AF from at least one source, and converted to a percent.

Data are limited to participants enrolled in Medicare fee-for-service.

To explore the impact of including incident AF cases ascertained only in CMS surveillance data, an analysis of the association between incident AF and the primary risk factors (age, male sex, white race, BMI, hypertension, diabetes, current smoking and prior heart disease) was performed. The beta estimates were almost identical and not significantly different in a model based exclusively on active ARIC follow-up methods compared to a model with the addition of cases from CMS surveillance (Table 4) suggesting the omission of CMS ascertained (mostly outpatient) AF does not bias the associations derived from active ARIC surveillance alone.

**Table 4:** Beta coefficients for the association of primary risk factors with incident atrial fibrillation (AF) using active Atherosclerosis Risk in Communities (ARIC) follow-up compared to active ARIC follow-up plus surveillance of Centers for Medicare and Medicaid Services (CMS) data.

	Active ARIC Follow-Up		Active ARIC Follow-Up and CMS Surveillance		
	Beta Coefficient	Standard Error	Beta Coefficient	Standard Error	p-value
Age, years	0.10	0.004	0.10	0.004	0.79
Female (Male)	−0.45	0.05	−0.47	0.04	0.75
Black (White)	−0.53	0.06	−0.57	0.06	0.70
BMI, kg/m^2^	0.05	0.005	0.04	0.004	0.79
Hypertensive (Normotensive)	0.40	0.05	0.38	0.05	0.83
Diabetic (Non diabetic)	0.43	0.06	0.41	0.06	0.78
Current smoker (Ever, never smoker)	0.63	0.05	0.59	0.05	0.58
Prior heart disease* (No prior heart disease)	0.67	0.07	0.67	0.06	0.96

Estimates correspond to log(hazard ratios) from Cox proportional hazards regression models.

ARIC =  Atherosclerosis Risk in Communities.

CMS =  Centers for Medicare and Medicaid Services.

Exposed (Referent).

*Prior heart disease defined as the presence of heart failure, myocardial infarction or coronary heart disease.

P-values from a one-degree-of-freedom Wald chi-square test of the null hypothesis that the beta coefficient from model one equals the beta coefficient from model two.

## Discussion

In this community-based prospective study, AF incidence rates were slightly lower based on active ARIC follow-up compared to CMS surveillance. The rates by either method followed a similar pattern, increasing with age and consistently higher among whites and men compared to blacks and women, respectively. Concordance of incident AF between data sources was very good, [14] although 19% more AF cases were identified from CMS largely due to outpatient ascertainment of AF. Furthermore, there appeared to be little bias in associations based only on active ARIC follow-up versus surveillance including CMS. To our knowledge, this is the first study to compare AF rates as well as concordance of diagnosis between data sources using only inpatient, only outpatient, and combined inpatient and outpatient data [7].

Reliance exclusively on active ARIC follow-up identified 831 incident cases of AF while CMS surveillance yielded 1,026 incident AF events. Concordance between the two data sources was good with a Κ statistic of 0.77 (95% CI: 0.75–0.80). As expected, because active ARIC follow-up relies exclusively on inpatient claims to identify AF, concordance improved when comparing only inpatient data. However, discrepancies persisted between the two data sources. Potential reasons for the discrepancies include that, among the 63 ARIC participants with AF ascertained from inpatient CMS data but not ARIC data, some participants stopped participating in annual telephone follow-up for the ARIC study but continued to be followed by the ARIC study for fatal events. Consequently, ARIC could not identify hospitalizations for these participants occurring outside of the geographic catchment area of the four ARIC communities. Possible reasons AF was obtained in ARIC data but not in CMS data include that the participant was admitted at a Veterans Affairs Hospital where CMS does cover the stay and that ARIC data captures up to 26 diagnosis and procedure codes while CMS MedPAR records only include 10 diagnosis and 6 procedure codes.

There are advantages and disadvantages of both active cohort follow-up and CMS surveillance to identify incident AF. Advantages of utilizing active cohort follow-up include ascertainment of AF at younger ages (prior to Medicare eligibility) and ability to identify AF regardless of type of insurance; disadvantages of this approach include missing outpatient diagnoses of AF and reliance on participants to report hospitalizations that occur outside of the study catchment area. The benefits of CMS surveillance data include detection of outpatient AF diagnoses and diagnoses for participants who stopped participating in cohort follow-up; disadvantages include lack of information on those <65 years as well as incomplete claims during Medicare Advantage enrollment.

Despite these opposing advantages and disadvantages, the results from the two methods were similar with comparable incidence rates, high concordance, and little evidence of bias of associations between AF and risk factors. These results can be interpreted several ways: supporting the exclusive use of active cohort follow-up, providing caution about the completeness of data from reliance on one method, and finding that two very different methods of AF ascertainment yielded similar results.

This study has several limitations. Medicare Advantage plans are not required to submit claims on their beneficiaries; a total of 18,194 person-years (19%) of follow-up were unobservable as a result of HMO enrollment (all other eligibility criteria were met) out of 97,740 total person-years. More importantly, person-years missed varied by center (Forsyth County, NC: 7,935, Jackson, MS: 1,640, Minneapolis, MN: 7,985, and Washington County, MD: 634). This makes use of CMS alone impractical for ARIC follow-up. Although exclusion of participants with Medicare Advantage limits the generalizability of the study findings, the concordance comparisons are applicable to the FFS population. ARIC involves whites and blacks from only three and two communities, respectively, and might not be generalizable to all whites and blacks in the US. In active ARIC follow-up, AF ascertainment relies primarily on hospital discharge codes and the diagnosis is not otherwise validated. However, this method has been found to have acceptable validity; in a sample of 125 hospital discharge summaries with a first ICD-9 code for AF, 89% were confirmed based on ECGs performed during that hospitalization [8]. Finally, neither data source identified AF using a gold standard and consequently high concordance between the two data sources supports, but does not prove, validity of these approaches.

The present study also has several strengths. First, its large sample size, with a substantial black population, and long follow-up of study participants enabled race-specific calculations. Most previous studies have been conducted in predominately white populations which is a limitation because some measures of validity, including PPV, are highly influenced by the prevalence of the disease in the source population, and blacks are known to have a lower risk of AF [8], [18]–[20]. Second, only one prior study has assessed the ability of administrative data, compared to physician reviewed hospital discharge summaries with a first ICD-9 code for AF and ECGs, to identify incident AF events; the PPV for AF was 89% and for incident AF was 62% [8]. In the present study, concordance of prevalent AF was similar to that of incident AF diagnosis (data not shown). The ability to identify incident AF events is especially important for comparative effectiveness research, studies of healthcare utilization over the entire disease course of AF, and drug safety surveillance; for example, a comparative effectiveness research study might want to include only treatment-naïve participants in order to decrease biases associated with treatment effectiveness in observational studies. Third, claims data are limited with respect to clinical characteristics since their primary purpose is for reimbursement. In this study, the ARIC data were linked to CMS data and, as a result, information not available in claims data, such as detailed and validated demographic, behavioral and comorbid conditions measured using standardized methodology, were present and included in descriptive analyses.

In conclusion, this study provides support for the potential value of utilizing multiple data sources to identify incident AF and suggests the need for caution about completeness of each data source. Nonetheless, two very different approaches to identifying incident AF produced similar results. Each approach has unique strengths and limitations and, when combined, could provide a more complete picture of newly-diagnosed AF. Moving forward, ARIC and similar studies should evaluate how to incorporate Medicare and other administrative data in the ascertainment of outcomes, factoring in the data limitations regarding coverage and quality.
